# A new species of armored scale, *Mycetaspis ailynaomi* (Hemiptera, Diaspididae, Aspidiotinae), associated with *Mammea americana* L. (Malpighiales, Calophyllaceae) from Puerto Rico

**DOI:** 10.3897/zookeys.108.1214

**Published:** 2011-06-17

**Authors:** Ramón A. Dones, Gregory A. Evans

**Affiliations:** 1 USDA/APHIS/PPQ PO Box 660520 Miami, Florida, USA, 33266; 2 USDA/APHIS/BARC West, Bldg. 005, Rm. 09A, Beltsville, Maryland, USA, 20705

**Keywords:** Sternorrhyncha, Diaspididae, Caribbean, Puerto Rico, *Mycetaspis*, new species

## Abstract

A new species of armored scale, *Mycetaspis ailynaomi* Dones and Evans is described and illustrated from specimens collected on mamey (*Mammea americana*) from Puerto Rico. A key to the species of *Mycetaspis* is provided.

## Introduction

Mamey (*Mammea americana* L., Calophyllaceae), also known as mammee apple, Santo Domingo apricot or South American apricot, is an evergreen, native to the West Indies and northern South America, whose fruit is edible. Mamey is confined to tropical or subtropical climates due to its sensitivity to low temperatures and seems remarkably resistant to pests and diseases. It has been introduced successfully into several tropical areas of the Old World (West Africa, Madagascar, southern Asia, Java, Philippines and Hawaii) but has not survived well in California and Florida (Morton 1987). It has formed part of the diet of the inhabitants of the Caribbean Islands for many generations. Mamey produces toxins that have medicinal and insecticidal properties and may cause discomfort, especially to the digestive system, in some individuals.

Nine species of armored scales have been reported from Puerto Rico on mamey. Martorell (1981) reported *Aspidiotus destructor*, *Howardia biclavis*, *Mycetaspis personata* and *Pseudaulacaspis pentagona*. Colón-Ferrer and Medina-Gaud (1998) reported *Abgrallaspis cyanophylli*, *Hemiberlesia palmae*, *Howardia biclavis*, *Mycetaspis personata*, *Selenaspidus articulatus*, and *Lopholeucaspis cockerelli*. In addition to these, Houser (1918) reported *Melanaspis calura* from Cuba and Deitz and Davidson (1986) listed *Melanaspis smilacis* as occurring on mamey but did not state in which country it was found on this host. Specimens of a new species of armored scale (Hemiptera: Diaspididae) of the genus *Mycetaspis* were found on mamey fruits seized by USDA/APHIS officials at the International Airport in San Juan, Puerto Rico during a pre-flight inspection.

The genus *Mycetaspis* Cockerell, 1897, currently comprises eight species (Ben-Dov 2010). The species in the genus are only known to occur in the Neotropical region and/or the Nearctic with the exception of *Mycetaspis personata*, which now occurs throughout much of the world. The genus, like most members of the subfamily Aspidiotinae, has the pygidium with macroducts of the 1-barred type, the second pygidial lobe not bilobulate, fringed plates present between the lobes, and the anterior and posterior spiracles without associated disk pores (Ferris 1941), except for *Mycetaspis bezerrai* (Arruda 1972). It is similar to the genus *Melanaspis* in that it has elongated paraphyses arising from the basal angles of the lobes, and between the 2nd and 3rd lobes, but differs from that genus in that the adult female has the frontal area sclerotized, raised and narrowing abruptly or rounded.

## Materials and methods

We follow the terminology used by Miller and Davidson (2005) and Watson (2002). The length of the pygidium was measured on the dorsal surface along the midline between the basal border and the bases of the median lobes. The abbreviations L1, L2, L3 and L4 stand for median, second, third and fourth pygidial lobes.

## Taxonomy

### Mycetaspis ailynaomi

Dones and Evans sp. n.

urn:lsid:zoobank.org:act:13FE4406-5E5B-4E18-A31C-8A4E252C795E

http://species-id.net/wiki/Mycetaspis_ailynaomi

Figs 1– [Fig F2] 6 

#### Adult female.

Appearance in life was not recorded, but the scale is not pupillarial. Body 1241µm long and 1136µm wide in the holotype; 1347µm long and 998µm wide in the paratype; almost circular. Pygidium slightly produced, almost (1.1 times) as broad as long, 279µm long by 423µm wide and 263µm long by 440µm wide in holotype and paratype, respectively.

#### Description.

**Cephalothorax.** Anterior margin of head heavily sclerotized with 10–14 tooth-like, sclerotized lobular processes. Eyes are represented by a sclerotized dot. Antennae each composed of a conspicuous seta and a tubercle. A group of 18–20 microducts in front of each anterior spiracle. A band of microducts between the anterior and posterior spiracles extending outward from the median area to the margin in a slight upward angle without reaching the margin. **Pygidium. Lobes.** With 4 well-developed lobes (L1-L4); L1 more or less symmetrical, longer than L2-L4, flask-shaped, divergent on the mesal margin, which are shorter than the lateral margin. L2 with mesal margin one third as long as the lateral margin, with 2 or 3 small round teeth. L3 and L4 similar to L2, but more diagonally set with the lateral margin about 4 times as long as the mesal margin. **Basal sclerosis.** Similar in shape to a paraphysis, arising from the mesal margin of the L1 lobes, about twice as long as the lobe and about one third as wide as the base of the lobe, almost parallel-sided and rounded on the top. **Paraphyses.** Arranged 2–3-3 on each side of the pygidium. First interlobular space (space between L1 and L2) with a long paraphysis terminating in a club and almost twice as long the basal sclerosis associated with L1; a smaller paraphysis arising from mesal base of L2 and slightly shorter than half the length of the long paraphyses in the space. Second interlobular space with 3 paraphyses: the mesal one arising from the lateral basal corner of L2, similar to the paraphysis arising from the mesal corner of L2 in size and shape; the median paraphysis in the space about twice as long as the mesal paraphysis, approximately the same as the long paraphysis in the first interlobular space in size and shape; the lateral paraphysis from the mesal corner of L3, similar to the mesal paraphysis. The third interlobular space has 2 or 3 paraphyses: a short paraphysis arising at the lateral basal corner of L3, followed by a longer one more than twice as long as the former one. Paraphyses arising from the mesal basal corner of L4 faint or almost obsolete; pygidial margin anterior to L4 also with some short paraphyses. **Plates.** Plates occurring between lobes, but their numbers are difficult to determine in the available specimens. Plates occurring between L1 and L2 slender  and simple, short, not extending beyond the apices of the lobes; plates between L2 and L3 slightly longer and wider with truncate apices; space between L3 and L4 appearing to have 3 short plates, one slender and 2 wider with truncate apices. **Anal opening.** Small, 14.5 µm in diameter, separated from the bases of L1 by a space about 6.5 times as long as its diameter. **Perivulvar pores.** Absent.

#### Male.

Unknown.

#### Type material.

Two adult females, holo- and paratype, Puerto Rico: 27.vi.2006, M. Resto, on *Mammea americana* fruit. Specimens are mounted in Canada Balsam. Both specimens are deposited in the U.S. Museum of Natural History (USNM) in Beltsville, Maryland.

#### Diagnosis.

*Mycetaspis ailynaomi* is most similar to *Mycetaspis defectopalus* Ferris in the shape of the pygidial lobes and the relative lengths and shapes of the paraphyses, but differs from the latter and other species in the genus in having 10–14 sclerotized lobular processes along the anterior margin of the cephalothorax; whereas the anterior margin of the cephalothorax is sclerotized, but smooth and rounded in the other species.

#### Biology.

This species in only known to occur on *Mammea americana* fruit in Puerto Rico. Several embryos were present in both the holotype and paratype specimens.

#### Etymology.

The species name is the combination of the names of the first author’s daughters, Ailyn and Naomi, as a testimony of his love to them.

**Figures 1–4.**
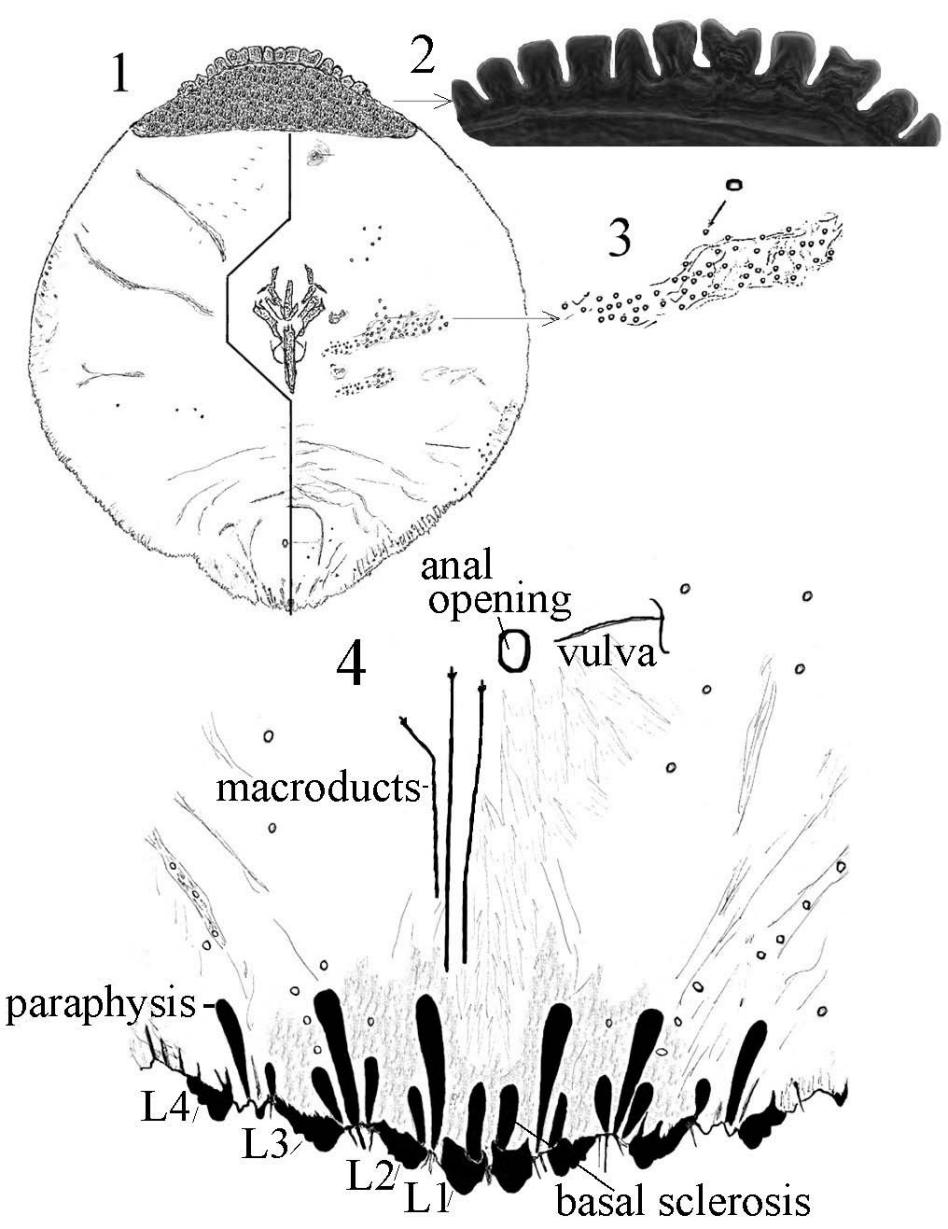
*Mycetaspis ailynaomi* holotype female **1** habitus **2** detail of lobes on head **3** detail of cluster of thoracic pores **4** detail of pygidial lobes.

**Figure 5. F2:**
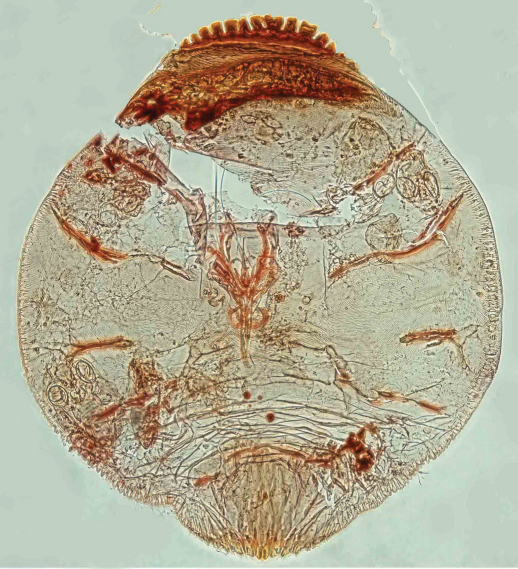
*Mycetaspis ailynaomi*, habitus of holotype female.

## Key to adult females of the genus *Mycetaspis*

**Table:** 

1	Perivulvar pores present; sclerotized area of anterior margin of head flatly rounded with a row of setae; Mexico, Guatemala, Panama, Venezuela	*Mycetaspis sphaerioides* (Cockerell)
1b	Perivulvar pores absent; sclerotized area of anterior margin of head produced without a row of setae	2
2(1b)	L1 each with an elongate, tapering basal sclerosis whose base is about as wide as the base of the L1 lobe	3
2b	L1 with the basal sclerosis narrow and arising from the mesal angle, its base is less than half as wide as the L1 base	4
3(2)	Perispiracular pores absent. L2 and L3 wider than long; L4 distinct; widespread	*Mycetaspis personata* (Comstock)
3b	Perispiracular pores present. L2 and L3 longer than wide; L4 merged into sclerotized margin; Brazil	*Mycetaspis bezerrai* Arruda
4(2b)	Eyes replaced with a thorn-like process; lateral area anterior to L4 with a series of relatively long paraphyses; Argentina, Brazil, Guyana, Mexico, Panama; USA (Texas)	*Mycetaspis apicata* (Newstead)
4b	Eyes not replaced with a thorn-like process; area anterior to L4 without a series of relatively long paraphyses (short paraphyses present in *Mycetaspis ailynaomi*)	5
5(4b)	Sclerotized area on anterior margin of head with a row of 10–14 large, protruding sclerotized lobular processes; area anterior to L4 with a series of short paraphyses; Puerto Rico	*Mycetaspis ailynaomi* Dones & Evans, sp. n.
5b	Head smooth, with no processes: area anterior to L4 without a series of short paraphyses	6
6(5b)	Anterior margin of head rounded, not incised, smoothly joining the lateral margin of the cephalothorax; Belize, Ecuador, Mexico, Nicaragua, Panama, Peru, USA (Florida, Texas)	*Mycetaspis defectopalus* Ferris
6b	Anterior margin of head, incised on each side of the apex, giving it a 3-lobed appearance, abruptly joining the lateral margin of the cephalothorax; Brazil	*Mycetaspis juventinae* Lepage & Giannotti

### Species not included in the key

*Mycetaspis brasiliensis* Hempel was described from Brazil. We do not have an illustration or specimens of this species to compare with the other species. According to Hempel (1932) it is similar to *Mycetaspis personata* but has a larger sclerotized area on the head, 4 pygidial lobes, and 18–20 pairs of paraphyses.

*Mycetaspis eneideae* Arruda was described from Brazil. No specimens of this species are available to us and the original illustration lacks sufficient details helpful for placing it in the key. Based on the original illustration of the species in  Arruda (1976), it appears that all of the pygidial lobes are fused together; there are no paraphyses that can be discerned.

**Figure 6.**
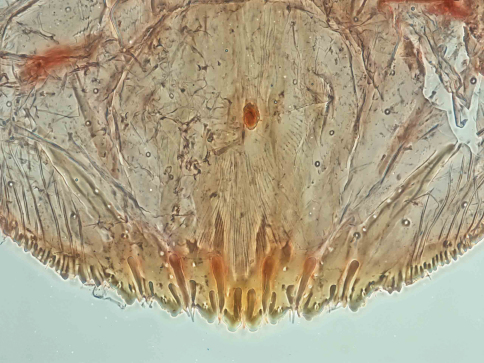
*Mycetaspis ailynaomi*, detail of pygidial lobes of holotype female.

**Figure 7.**
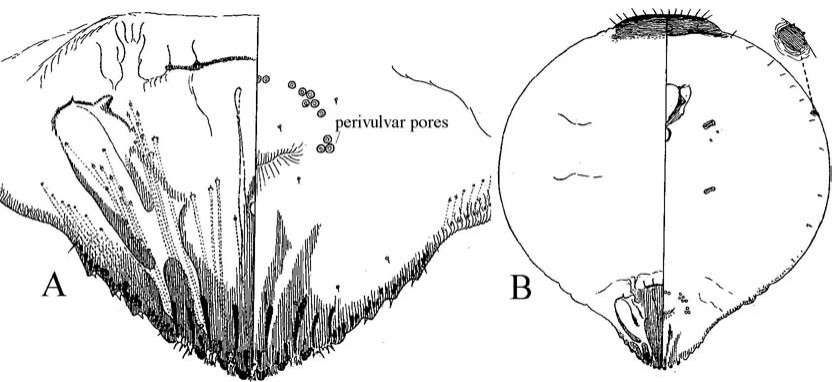
*Mycetaspis sphaerioides* female **a** pygidium **b** habitus (after Ferris 1941).

**Figure 8.**
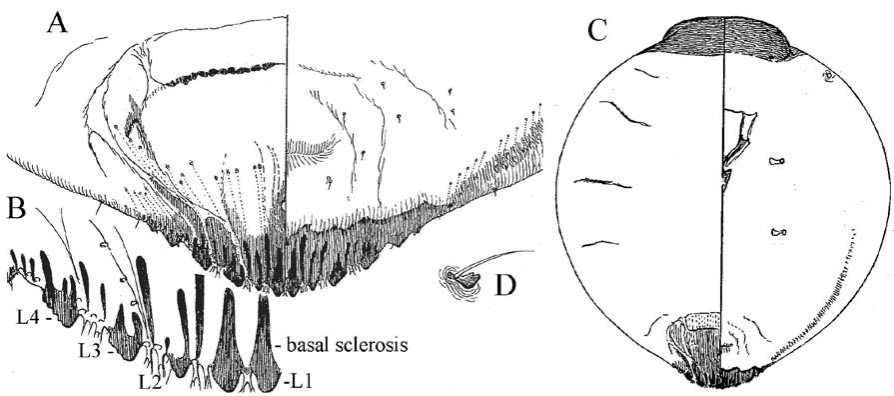
*Mycetaspis personata* female **a** pygidium **b** detail of pygidium, c) habitus,  d) antenna (after Ferris 1941).

**Figure 9.**
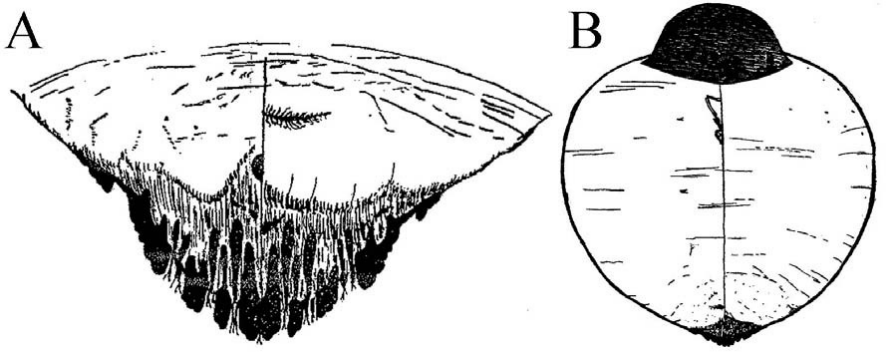
*Mycetaspis bezerrai* female **a** pygidium **b** habitus (after Arruda 1972).

**Figure 10.**
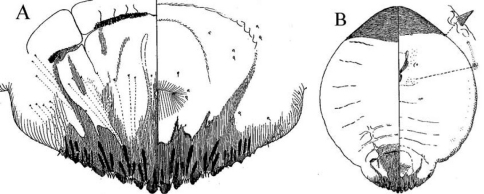
*Mycetaspis apicata* female **a** pygidium **b** habitus (after Ferris 1941).

**Figure 11.**
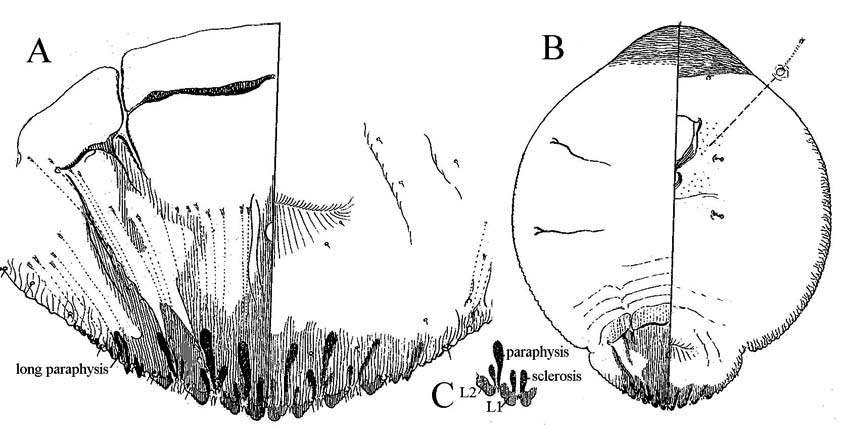
*Mycetaspis defectopalus* female **a** pygidium **b** habitus **c** detail of L1 and L2 lobes (after Ferris 1941).

**Figure 12.**
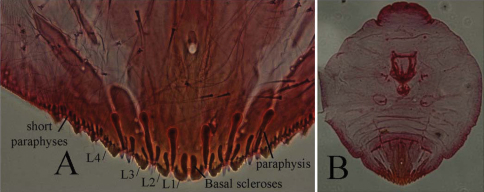
*Mycetaspis juventinae* female (topotype) **a** pygidium **b** habitus.

## Acknowledgements

The first author thanks Mr John W. Dooley, my mentor in the study of the armored scales and, the late Dr Leonce Bonnefil, who introduced me to Entomology. I also thank the San Juan, Puerto Rico Inspection Station where the scale was intercepted and to the Miami Inspection Station for funding this publication. The views and ideas expressed herein are not necessarily those of the USDA.

## Supplementary Material

XML Treatment for Mycetaspis ailynaomi
